# Treatment of Acute Ulcerative Colitis with Zinc Hyaluronate in Mice

**DOI:** 10.4014/jmb.2408.08050

**Published:** 2025-02-14

**Authors:** Lan Zhang, Xuedan Fu, Jiazheng Li, Wan Xiao, Xi Xiong, Huixia Lv, Zhenhai Zhang, Jianming Ju

**Affiliations:** 1 Affiliated Hospital of Integrated Traditional Chinese and Western Medicine, Nanjing University of Chinese Medicine, Nanjing 210023, P.R. China; 2 Jiangsu Province Academy of Traditional Chinese Medicine, Nanjing 210023, P.R. China; 3 School of Medicine, Nanjing University of Chinese Medicine, Nanjing 210023, P.R. China; 4 Department of Pharmaceutics, School of Pharmacy, China Pharmaceutical University, Nanjing 211198, P.R. China

**Keywords:** Zinc hyaluronate, sodium hyaluronate, ulcerative colitis, intestinal flora, short-chain fatty acids

## Abstract

Ulcerative colitis (UC) is a type of inflammatory bowel disease arising from numerous factors, while UC patients face insufficient treatment options and a high incidence of adverse reactions to the current therapies. As a functional food additive, hyaluronic acid plays a certain role in intestinal repair. In this study, we constructed a mouse model of dextran sulfate sodium (DSS)-induced UC to examine the effects and underlying mechanisms of action of zinc hyaluronate (ZnHA) on the pathogenesis of UC. ZnHA effectively alleviated key clinical UC symptoms, such as weight loss, loose stools, and bloody stools. Mechanistically, ZnHA attenuated the expression of inflammatory factors, such as tumor necrosis factor-α, interleukin (IL)-6, and myeloperoxidase while upregulating the expression of IL-10. Furthermore, through intestinal flora and short-chain fatty acid analyses, ZnHA was found to promote propionic acid production by enriching beneficial bacteria. ZnHA simultaneously enhanced the expression of tight junction proteins, specifically ZO-1 and occludin, thereby restoring intestinal barrier function. Overall, our findings elucidate the therapeutic potential of ZnHA in treating acute UC by inhibiting intestinal inflammation and regulating flora, while also providing further theoretical support for development of hyaluronic acid to treat this disease.

## Introduction

Ulcerative colitis (UC) is a globally prevalent disease that primarily affects adults aged 30–40 years [1]. UC is characterized by lesions extending from the rectum to the proximal segment of the colon [2]. Patients with UC frequently experience intestinal mucosal ulcers, rectal bleeding, diarrhea, and abdominal pain [3], which significantly compromise their overall quality of life. The pathogenesis of UC is primarily associated with genetic and environmental factors in addition to immune disorders of the intestinal mucosa [4]. Without intervention, the risk of UC progressing to colorectal cancer increases substantially, which has put this disease under intensified focus [5]. Currently, mesalazine, corticosteroids, immunomodulators, and biological agents are the mainstays of UC treatment [6-8]. Mesalazine is widely recognized as the first-line treatment for mild to moderate UC, exhibiting excellent tolerability but with limited efficacy, whereas corticosteroids and other similar medications have certain side effects [9, 10].

Numerous studies have highlighted the crucial role of maintaining intestinal microbial balance in the progression of colitis. Based on this foundation, approaches such as probiotics [11, 12] or fecal microbiota transplantation (fMT) [13, 14] have emerged as potential therapies for UC. Additionally, during the fermentation process of certain intestinal bacteria, beneficial metabolites known as short-chain fatty acids (SCFAs), including acetate, propionate, and butyrate [15], are produced in the colon, and these metabolites have demonstrated significant therapeutic effects in treating inflammatory bowel diseases, such as colitis [16]. Moreover, bacteria including *Bifidobacterium bifidum*, *Propionibacterium freudenreichii*, and *Clostridium butyricum* can significantly enhance the production of acetic, propionic, and butyric acids in mice with inflammatory bowel disease [17], thereby effectively modulating UC. fMT, as an effective treatment for UC, primarily alleviates UC symptoms by altering intestinal permeability, albeit with associated side effects and high costs. Therefore, there is an urgent need to develop a safe, effective, and low-cost therapeutic drug for UC.

Hyaluronic acid (HA), as a linear polysaccharide with mucosal adsorption properties, is widely used in various fields, such as food, pharmaceuticals, and cosmetics, with exceptional biocompatibility. Additionally, studies have revealed the remarkable anti-inflammatory properties of HA; thus, it is often used to alleviate adverse reactions affecting the eyes, joints, and skin [18-20]. Upon entering the intestine, HA is capable of absorption by the intestine following degradation by intestinal microorganisms, and is subsequently transported throughout the body via the blood and lymphatic system [21]. Notably, HA plays a pivotal role in wound healing and enhancing intestinal barrier function [22, 23]. Furthermore, sodium hyaluronate (NaHA)-based drugs can maintain epithelial barrier integrity and intestinal homeostasis in mice with UC by inhibiting inflammation and modulating the intestinal microbiota [24, 25].

As another salt form of HA, zinc hyaluronate (ZnHA) has excellent antibacterial and fungicidal properties [26] and can improve the adhesion properties of ophthalmic products [27]. This is attributed to the crucial role of zinc ions in the wound repair process [28]. In addition, zinc demonstrates a remarkable capacity to promote tissue repair while exerting anti-inflammatory effects, granulation promotion, re-epithelialization, and extracellular matrix remodeling [29]. We propose that with ZnHA, the synergistic effect of HA and zinc, coupled with the combined anti-inflammatory and repair properties, may facilitate the recovery of UC through distinct mechanisms.

In this study, we hypothesized that ZnHA could ameliorate dextran sulfate sodium (DSS)-induced acute UC in mice. To validate the therapeutic efficacy of ZnHA and explore its underlying mechanisms, we conducted multiple rounds of research. First, we investigated the *in vitro* anti-inflammatory effects of ZnHA using mouse RAW264.7 macrophages, and we then tested the therapeutic effects of ZnHA in a DSS-induced acute UC mouse model. Subsequently, we examined the efficacy of ZnHA in repairing the intestinal barrier by detecting the expression of tight junction proteins. Finally, we employed 16S rRNA sequencing technology and gas chromatography-mass spectrometry (GC-MS) to study the impact of ZnHA on the intestinal microbiota and SCFAs in mice with UC, respectively.

## Materials and Methods

### Materials and Reagents

ZnHA and NaHA were purchased from Bloomage Biotechnology Co., Ltd. (China). Rabbit polyclonal antibodies against ZO-1 and occludin and a mouse monoclonal antibody for β-actin were purchased from Wuhan Proteintech (China). DSS (MW: 36000-50000 Da) was purchased from MP Biomedicals (USA).

A Cell Counting Kit-8 (CCK-8) was provided by Shanghai Yeasen BioTechnology Co., Ltd. (China). Enzyme-linked immunosorbent assay (ELISA) and myeloperoxidase (MPO) kits were provided by Nanjing Jiancheng Biological Institutions (China). The ntric oxide (NO) assay kit was provided by Beyotime Institute of Biotechnology (China).

### Cell Experiments

Mouse mononuclear macrophage RAW264.7 cells were cultured in Dulbecco's Modified Eagle Medium (Keygen BioTECH, China), with 10% fetal bovine serum (Biological Industries, Kibbutz Beit-Haemek, Israel). RAW264.7 cells in the logarithmic growth phase were cultured with 0–200 μg/ml ZnHA and 0–3,000 μg/ml NaHA for 24 h. The toxicity of the drug to cells was detected by adding a CCK-8 solution.

Next, lipopolysaccharide (LPS) was used to trigger inflammation in RAW264.7 cells. Subsequently, ZnHA and NaHA were added separately. Cell supernatants were collected for NO detection.

### Animal Experiments

Eight-week-old male BALB/c mice (weight: 20 ± 2 g) were obtained from Yangzhou University (animal license no. SYXK [Su] 2022–0009, China). All animals were kept under standard laboratory conditions with an ambient temperature of 22–24°C and relative humidity of 55 ± 5% under a 12-h light-dark cycle with unlimited access to food and water. All experiments were performed according to the criteria approved by the Animal Ethics Committee of the Jiangsu Provincial Academy of Chinese Medicine (AEWC-20230313-275).

All mice were acclimatized for 10 days prior to the initiation of the experiment. All mice in the formal experiment were randomly divided into control (Ctrl), DSS, NaHA, and ZnHA groups, with six mice in each group. Starting from the first day of the experiment, except for the Ctrl group, other mice drank 2.0% DSS solution freely for 7 days for acute UC. Intragastric administrations were conducted daily, lasting for 8 days from the onset of modeling. The Ctrl and DSS groups were administered normal saline by gavage at a dose of 10 ml/kg. The NaHA and ZnHA groups were administered 50 mg/kg by gavage. During the experiment, the diet, activity, and hair status of the mice were observed daily. Body weight, stool traits, and hematochezia were recorded daily. At the same time, the disease activity index (DAI) of mice was calculated. The DAI score was determined on the basis of the degree of weight loss, loose stools, and bloody stools [13]; the specific scoring criteria are shown in Table 1. On day 9, all mice were euthanized.

### Simultaneously, the colon was obtained, and stool in the cecum was collected for intestinal microbiome analysis and SCFA analysis [30]. After measuring the colon length, a quarter of the colon was fixed in 4% (v/v) formalin, and the remaining samples were stored at -80°C for subsequent analysis of other indicators. Additionally, the collected spleens were weighed, and the spleen index was calculated in accordance with the following formula [31]:

Spleen index (mg/g) = spleen weight (mg) / final day weight (g).

### Hematoxylin and Eosin (H&E) Staining

After fixation for 24 h, the colons were dehydrated, embedded, sectioned, and stained with H&E. Then, a light microscope (Olympus CX31; Olympus, Japan) was used to observe the slices for pathological alterations of the colons.

### Detection of Inflammatory Cytokinesis

Some colon proteins were extracted by homogenizing with phosphate-buffered saline, and cytokines (TNF-α, IL-6, IL-10, MPO) in the colon were determined using the ELISA reagents and standard kit. All experimental procedures were performed in accordance with the manufacturer's guidelines.

### Western Blot Analysis

The remaining colon proteins were extracted using RIPA buffer containing 1% phenylmethanesulfonyl fluoride. After separating the same amount of protein using 8% sodium dodecyl sulfate–polyacrylamide gel, the proteins were transferred to a polyvinylidene fluoride membrane (0.45 μm). The target band was then blocked and incubated with an antibody. Observation and imaging were performed using a gel imaging system (Tanon 5200Multi, China), and band grayscale values were calculated using ImageJ software.

### 16S rRNA Sequencing of Gut Microbiota

Total genomic DNA of microbial origin was isolated from the cecal contents of each group of mice using the FastPure Feces DNA Isolation Kit (Shanghai Major Yuhua, China). The quality and concentration of DNA were determined using a NanoDrop2000 spectrophotometer (Thermo Fisher Scientific, USA).

The primer pair 338F (5'-ACTCCTACGGGAGGCAGCAG-3') and 806R (5'-GGACTACHVGGGTWTCTAAT-3') was used to amplify the hypervariable region V3–V4 of the bacterial 16S rRNA gene. A PCR purification kit (YuHua, China) and a Qubit 4.0 (Thermo Fisher Scientific) were used for purification and quantification, respectively. The purified amplicons were combined in equimolar quantities and underwent paired-end sequencing using the Illumina PE300/PE250 platform (Illumina, USA).

The fastp [32] software (version 0.19.6) was used to perform quality control on paired-end raw sequencing reads, and the FLASH [33] software (version 1.2.11) was employed for sequence assembly. Subsequently, we used the UPARSE (version 7.1) [34] software to conduct operational taxonomic unit (OTU) clustering on the basis of 97% similarity for the quality-controlled and assembled sequences, and to remove chimeras. We annotated the OTU species taxonomy using the RDP classifier [35] (version 2.11) by aligning the information against the Silva 16S rRNA gene database (version 138) with a confidence threshold of 70%, and counted the community composition of each sample at different taxonomic levels. Next, we performed 16S functional prediction analysis using the PICRUSt2 [36] software (version 2.2.0). All data analyses were conducted on the Majorbio Cloud Platform (https://cloud.majorbio.com).

### Determination of SCFAs

Reference solutions of acetic acid, propionic acid, butanoic acid, isobutyric acid, valeric acid, isovaleric acid, hexanoic acid, and isohexanoic acid were prepared from 500 to 6,000 μg/ml with n-butanol. In this study, 1,000 μg/ml 2-ethylbutyric acid was used as the internal standard. A 20 mg stool sample was added to 800 μl of 0.5%phosphoric acid (containing 10 μg/ml of 2-ethylbutyric acid). Samples were cryomilled, sonicated, and centrifuged to separate the supernatant, and then n-butanol extraction was performed. After vortexing and low-temperature sonication, the supernatant was centrifuged for GC-MS analysis. Analysis was conducted using the Agilent 8890B-5977B GC/MSD system equipped with an HP FFAP capillary column (30 m × 0.25 mm × 0.25 μm, Agilent J&W Scientific, USA). MassHunter Quantitative Analysis software (version v10.0.707.0, Agilent) was employed to automatically detect and integrate the fragments of each ion of the SCFAs of interest and assist with manual examination. A quantitative determination of the concentration of SCFAs in fecal samples was achieved by calculating the detection concentration of each sample on the basis of a standard curve.

### Statistical Analysis

Statistical analysis of the experimental data was performed using GraphPad Prism 9.5 software (USA). Differences between groups were assessed using Student’s *t*-test. Spearman’s analysis was used for correlation analysis. A *p*-value of < 0.05 was considered to express a statistically significant difference.

## Results

### Effects of ZnHA on LPS-Induced Inflammation in RAW264.7 Cells

**Fig. 1:**
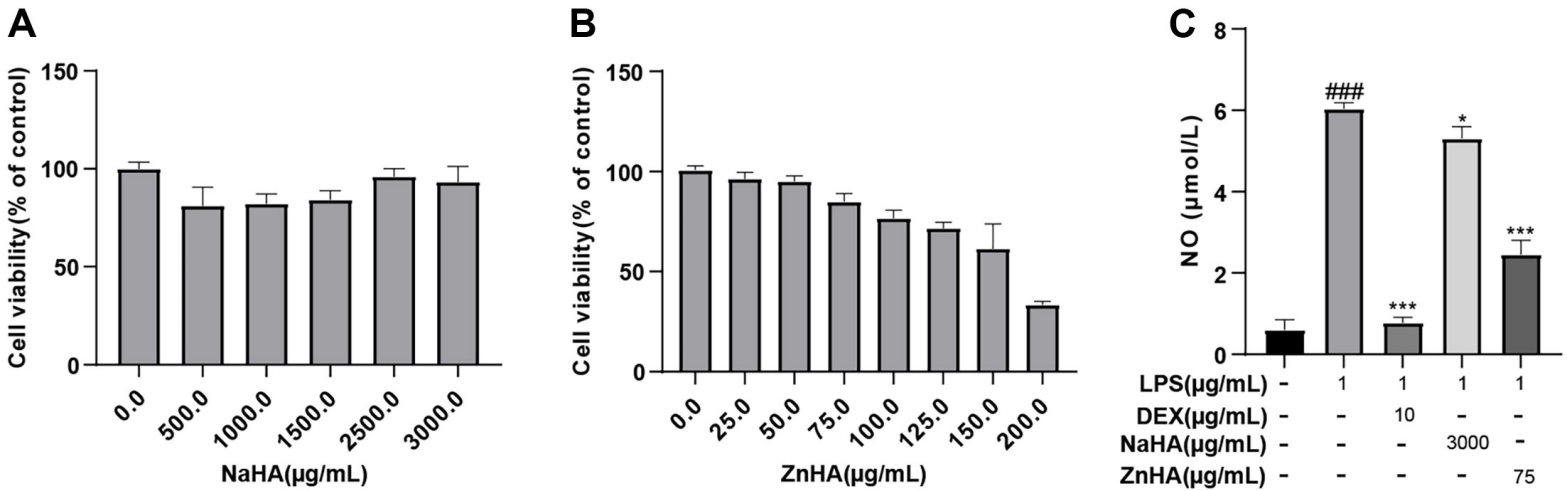
The effect of ZnHA on inflammation in RAW264.7 cells. Cytotoxicity of different concentrations of NaHA (**A**) and ZnHA (**B**) on RAW264.7 cells. Contents of NO (**C**) in RAW264.7 cells (*n* = 6). Data are presented as mean ± SD. ^###^*p* < 0.001, ^##^*p* < 0.01, ^#^*p* < 0.05 versus Ctrl group. ***p < 0.001, **p < 0.01, *p < 0.05 versus LPS group. Ctrl, control; LPS, lipopolysaccharide; DEX, dexamethasone; NaHA, sodium hyaluronate; ZnHA, zinc hyaluronate; NO, nitrous oxide; SD, standard deviation.

Cytotoxic concentrations of ZnHA and NaHA were measured to determine the safe concentration range for anti-inflammatory experiments. Based on the findings from cytotoxicity experiments, NaHA displayed no toxicity to RAW264.7 cells within the concentration range of 0–3,000 μg/ml (Fig. 1A). The toxicity of ZnHA to RAW264.7 cells within the dose range of 0–200 μg/ml was dose-dependent, and the toxicity was low within the dose range of 0–75 μg/ml, with cell viability maintaining above 75% at these dosages [37] (Fig. 1B).

Subsequently, the inflammatory cell model of RAW264.7 cells was induced by LPS, and the degree of inflammation was assessed by measuring the levels of NO. LPS-induced RAW264.7 cells were treated with 75 μg/ml ZnHA and 3,000 μg/ml NaHA for 24 h, respectively. ZnHA and NaHA significantly restored the LPS-induced increase in NO content (Fig. 1C). The recovery effect of ZnHA on NO was superior to that of NaHA. The results indicated that ZnHA inhibited the LPS-induced inflammatory response.

### ZnHA Alleviates the Symptoms of DSS-Induced UC in Mice

**Fig. 2:**
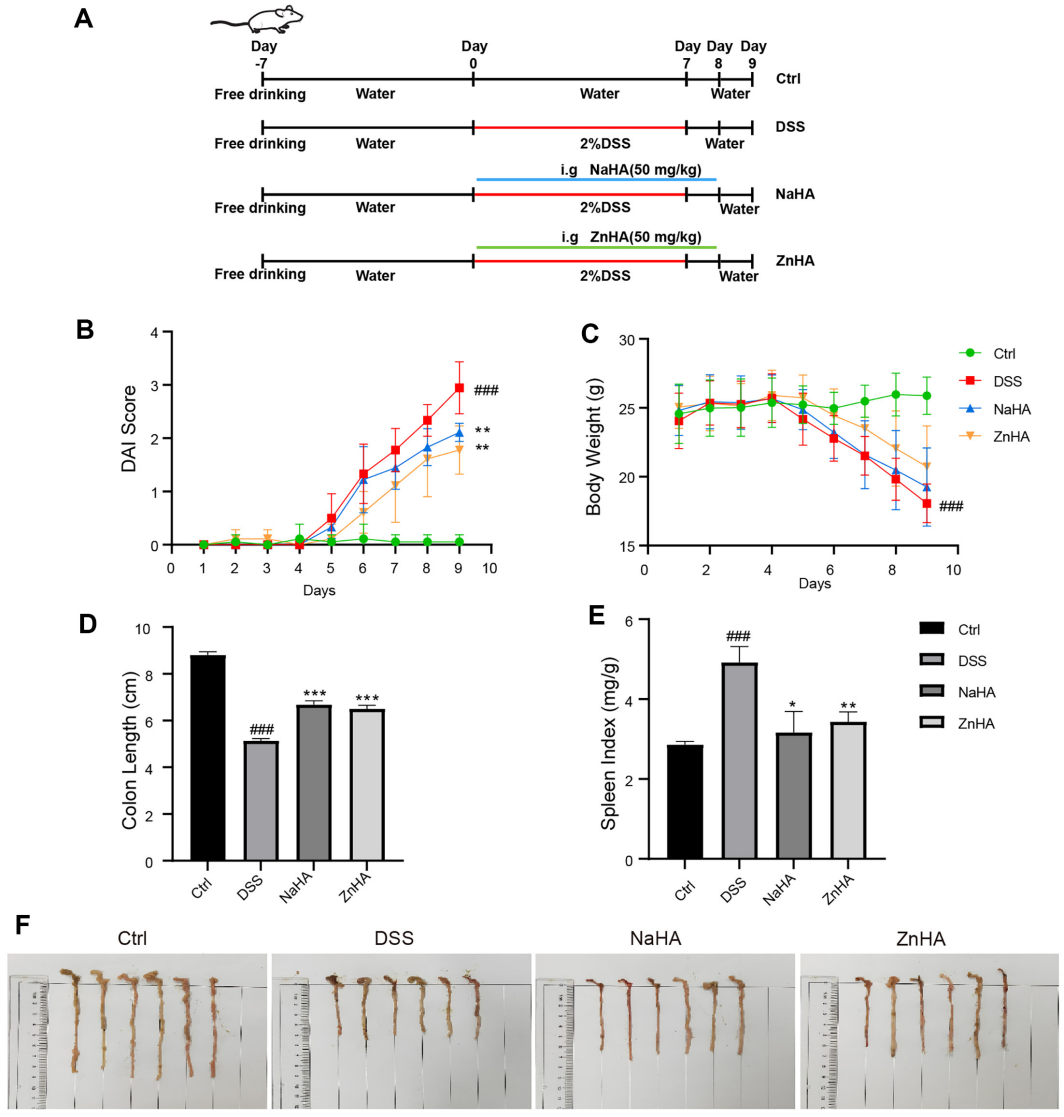
ZnHA and NaHA ameliorated DSS-induced acute UC in mice. Modeling and administration of experimental mice (**A**). The effects of different doses of ZnHA and NaHA on mice with UC: DAI scores (**B**); body weight (**C**); colon length (**D**); spleen index (**E**); images of the colon (F) (*n* = 6). The oral dosage for ZnHA and NaHA is 50 mg/kg. Data are presented as mean ± SD. ^###^*p* < 0.001, ^##^*p* < 0.01, ^#^*p* < 0.05 versus Ctrl group. ***p < 0.001, **p < 0.01, *p < 0.05 versus DSS group. NaHA, sodium hyaluronate; ZnHA, zinc hyaluronate; DAI, disease activity index; DSS, dextran sulfate sodium; Ctrl, control; SD, standard deviation.

Through preliminary experimental studies, we found that ZnHA and NaHA administered at a dose of 50 mg/kg demonstrated superior therapeutic effects in UC compared with a dose of 25 mg/kg (Fig. S1). On this basis, we orally administered 50 mg/kg ZnHA and 50 mg/kg NaHA for eight consecutive days at the same time as modeling to evaluate their efficacy in the treatment of UC (Fig. 2A). The results showed that animals in the DSS group started losing weight and began to have loose and bloody stools on the fifth day after drinking water freely with 2.0% DSS. As shown in Fig. 2B-2C, the DAI score in the DSS group gradually escalated and was significantly distinct from that of the Ctrl group, whereas the body weight of the DSS group notably decreased (*p* < 0.001). After administration of 50 mg/kg ZnHA or NaHA, the symptoms were significantly alleviated, and the DAI score was significantly reduced (*p* < 0.01).

The colon of each animal was observed, as shown in Fig. 2D and 2F. The colon lengths were significantly shortened after DSS modeling (*p* < 0.001). There were obvious bleeding points in the colon and perforations in some of the colon, and the colon length was notably restored after the administration of ZnHA or NaHA (*p* < 0.001). Furthermore, as shown in Fig. 2E, the spleen index significantly increased after DSS administration (*p* < 0.001). After administration of ZnHA or NaHA, the spleen index significantly decreased, and the final index was comparable to that of the Ctrl group. The changes in these indicators indicate that ZnHA or NaHA can significantly alleviate the symptoms of UC with a good therapeutic effect.

### ZnHA Inhibits Colon Injury

**Fig. 3:**
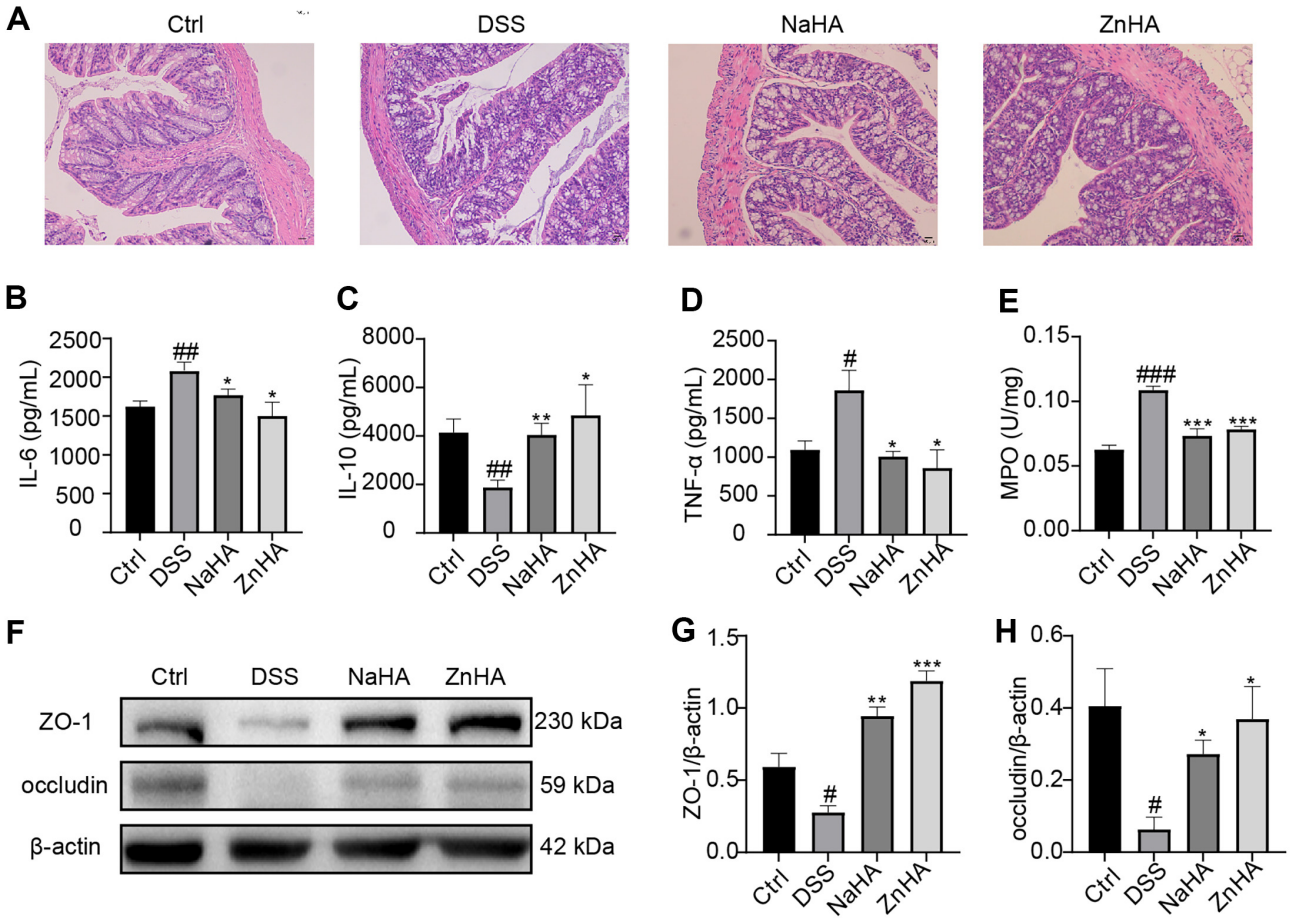
ZnHA inhibits colonic inflammation and injury in mice with UC. Hematoxylin and eosin (H&E) staining (100×) (**A**). Contents of cytokines related to colon inflammation: IL-6 (**B**), IL-10 (**C**), TNF-α (**D**), MPO (**E**) (*n* = 6). Expressions of tight junction proteins (ZO-1 and occludin) in colon tissues (**F**). Relative protein contents of ZO-1 (**G**) and occludin (**H**) (*n* = 3). Data are presented as mean ± SD. ^###^*p* < 0.001, ^##^*p* < 0.01, ^#^*p* < 0.05 versus Ctrl group. ***p < 0.001, **p < 0.01, *p < 0.05 versus DSS group. Ctrl, control; NaHA, sodium hyaluronate; ZnHA, zinc hyaluronate; TNF, tumor necrosis factor; DSS, dextran sulfate sodium; MPO, myeloperoxidase; SD, standard deviation.

At the same time, we performed H&E staining of the colon tissue to observe its pathological changes. As shown in Fig. 3A, the colon tissue of the Ctrl group had intact epithelial cells and crypt structures, lacking any evident signs of inflammatory infiltration. The colon tissue of the DSS group showed severe lesions, severely compromised epithelial cells, disappearance of a large number of goblet cells and crypts, and obvious inflammatory cell infiltration. After administration of ZnHA and NaHA, damage to the colonic mucosa and infiltration of inflammatory cells were alleviated, and crypts and goblet cells were restored. This indicates that ZnHA significantly repaired intestinal damage.

### ZnHA Inhibits the Inflammatory Response in Mice with UC

Inflammatory factors play crucial roles in the development of intestinal inflammatory diseases. IL-6 and TNF-α are typical pro-inflammatory cytokines, and IL-10 is a classical anti-inflammatory cytokine [13]. MPO is a marker associated with neutrophils that is used to evaluate inflammation in intestinal inflammatory diseases [38]. Here, we detected the inflammatory factors in the colon of all mice [39]. After the UC model was established using DSS, mice developed systemic inflammation. IL-6 and TNF-α levels in the DSS group were notably elevated compared with those in the Ctrl group (*p* < 0.01), and IL-10 levels were markedly lower than that in the Ctrl group (*p* < 0.01). After administration of ZnHA and NaHA, IL-6 and TNF-α levels were much lower than those in the DSS group (*p* < 0.05), and the IL-10 level was greater than that in the DSS group (*p* < 0.05) (Fig. 3B-3D). Next, we examined MPO expression. MPO levels in the DSS group were superior to those in the Ctrl group (*p* < 0.001), and MPO levels after administration of ZnHA and NaHA were inferior to those in the DSS group (*p* < 0.001) (Fig. 3E). The expression of these inflammatory factors demonstrated that ZnHA exerted remarkable inhibitory effects on DSS-induced colon inflammation. At the same time, the anti-inflammatory effect of ZnHA was slightly better than that of NaHA.

### ZnHA Promotes the Recovery of Tight Junction Proteins

Our findings indicated that both ZnHA and NaHA are effective in the treatment of UC. Therefore, we investigated whether they also exert an influence on the treatment of UC via the same mechanism of action. Occludin and ZO-1 are classic tight junction proteins. In DSS-treated colon tissue, the expressions of occludin and ZO-1 were markedly reduced, with almost no significant expression. As shown in Fig. 3F-3H, the amount of occludin and ZO-1 was appreciably restored by the administration of ZnHA and NaHA. These findings indicate that ZnHA and NaHA can effectively restore DSS-induced intestinal barrier damage. Simultaneously, the expressions of ZnHA were higher than those of NaHA, but there was no significant difference.

### Effects of ZnHA on Intestinal Flora in Mice with UC

**Fig. 4:**
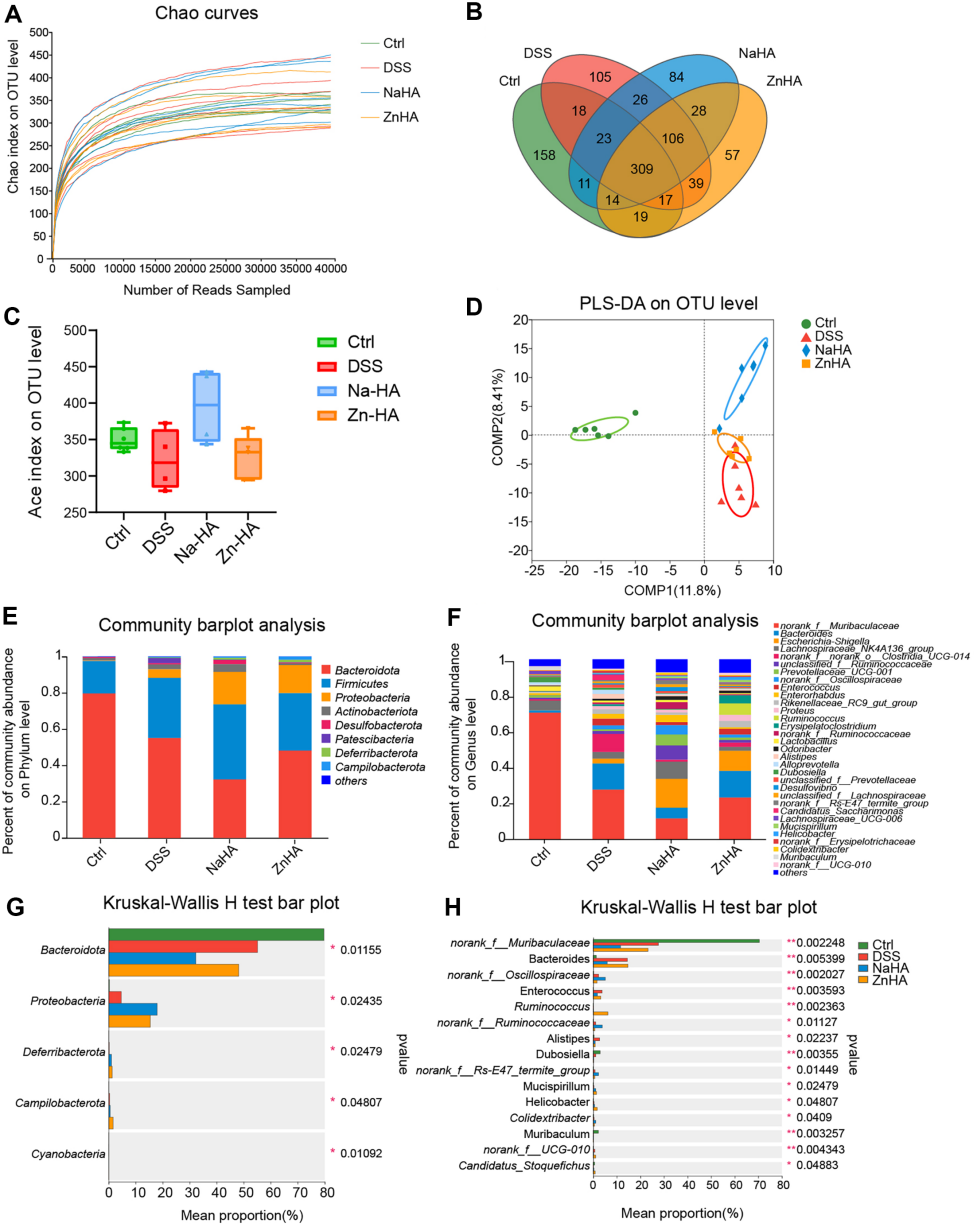
The effects of ZnHA on intestinal flora. Effect of ZnHA on colon microbes in mice with DSS-induced UC (*n* = 6). Chao curves of gut microflora metagenomes at the OTU level (**A**) Venn diagram of OTU (**B**) Ace index of OTUs (**C**) PLS-DA of bacterial beta-diversity (**D**) Community abundance at the phylum level (**E**). Community abundance at the genus level (**F**). Phylum with different abundance in each group (**G**) Genus with different abundance in each group (H) ZnHA, zinc hyaluronate; DSS, dextran sulfate sodium; PLS-DA, partial least squares discriminant analysis; OTU, Operational Taxonomic Unit.

Next, we analyzed the gut microbiota of each mouse using 16S rRNA gene sequencing [40]. As the number of sequences increased, the sparse curves of every sample tended to lean toward saturation, showing that the data were reliable and suitable for subsequent bioinformatic analysis [41] (Fig. 4A). Next, the assemblage between the groups was analyzed. The Venn diagram shows that the Ctrl, DSS, NaHA, and ZnHA groups contained 569, 643, 601, and 589 OTUs, respectively. There were 135 OTUs shared by all groups (Fig. 4B).

The results of α-diversity analysis revealed no significant difference in the Ace index among the groups, indicating that the variation in gut microbiota richness was minimal across all mice (Fig. 4C). Subsequently, partial least squares discriminant analysis was employed to investigate β-diversity, with the results presented in Fig. 4D. The gut microbiota structure of mice in the DSS group exhibited a clear separation from that of the Ctrl group. After ZnHA administration, the distance between the ZnHA-treated group and the Ctrl group was significantly shortened, indicating a higher similarity in bacterial communities between the ZnHA-treated and Ctrl groups.

In addition, we analyzed the composition of intestinal microbial communities in each group of mice. A total of eight phyla with abundances > 1% were found (Fig. 4E and 4G), including *Bacteroidota*, *Firmicutes*, *Proteobacteria*, *Actinobacteriota*, *Desulfobacterota*, *Patescibacteria*, *Deferribacterota* and *Campilobacterota*. Among these, *Bacteroidota*, *Firmicutes*, and *Proteobacteria* were the dominant phyla in the fecal microbiota. In comparison with results of the Ctrl group, the proportion of *Bacteroidota* decreased significantly after DSS modeling, and the proportions of *Firmicutes*, *Proteobacteria*, and *Patescibacteria* increased.

Furthermore, according to the analysis, 34 genera with an abundance > 1% were found (Fig. 4F and 4H), including *norank_f__Muribaculaceae*, *Bacteroides*, *Escherichia-Shigella*, and *unclassified_f__Ruminococcaceae*. At the genus level, the fecal microbiota was primarily made up of *norank_f_Muribaculaceae*, *Bacteroides*, *Escherichia-Shigella*, and *Lactobacillus*. After DSS modeling, *norank_f__Muribaculaceae* and *Lachnospiraceae_ NK4A136_group* decreased significantly and *Enterococcus* abundance increased significantly. The expression of *norank_f__norank_o__Clostridia_UCG-014* significantly decreased after NaHA and ZnHA administration.

The relative abundances of *Akkermansia*, *Helicobacter*, *Enterorhabdus*, *Parasutterella*, *Mucispirillum*, *Anaerotruncus* and other bacteria in the intestinal contents of the ZnHA group were the highest. The relative abundance of *Lachnospiraceae_NK4A136_group*, *unclassified_f__Ruminococcaceae*, *Prevotellaceae_UCG-001*, *norank_f__Ruminococcaceae* and other bacteria in the intestines of the NaHA group was the highest.

**Fig. 5:**
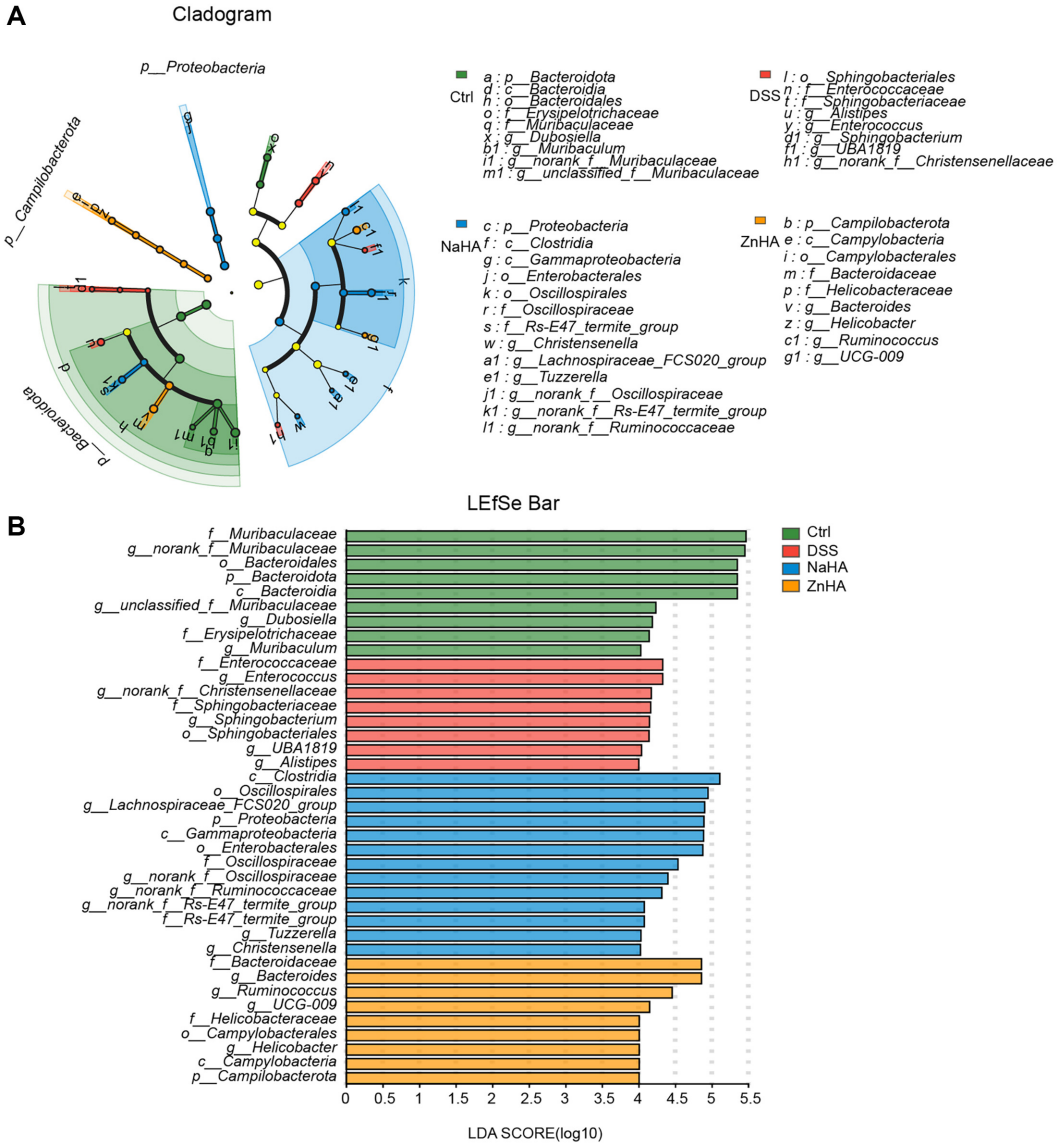
Advantageous microbiota regulated by ZnHA. LEfSe differential analysis (**A**) and LDA scores (**B**) of mice intestinal flora. DSS, dextran sulfate sodium; Ctrl, control; NaHA, sodium hyaluronate; ZnHA, zinc hyaluronate; LEfSe, linear discriminant analysis effect size; OTU, operational taxonomic unit.

In addition, we conduced linear discriminant analysis effect size (LEfSe) to identify key species in the occurrence and development of diseases by analyzing multilevel differential species [42]. LEfSe analysis (LDA threshold of 4.0) was conducted on four groups of samples. The dominant bacteria in the Ctrl, DSS, NaHA, and ZnHA groups were *f__Muribaculaceae*, *f__Enterococcaceae*, and *c__Clostridia*, *f__Bacteroidaceae*, respectively, as shown in Fig. 5A-5B.

### Effects of ZnHA on SCFAs in Mice with UC

**Fig. 6:**
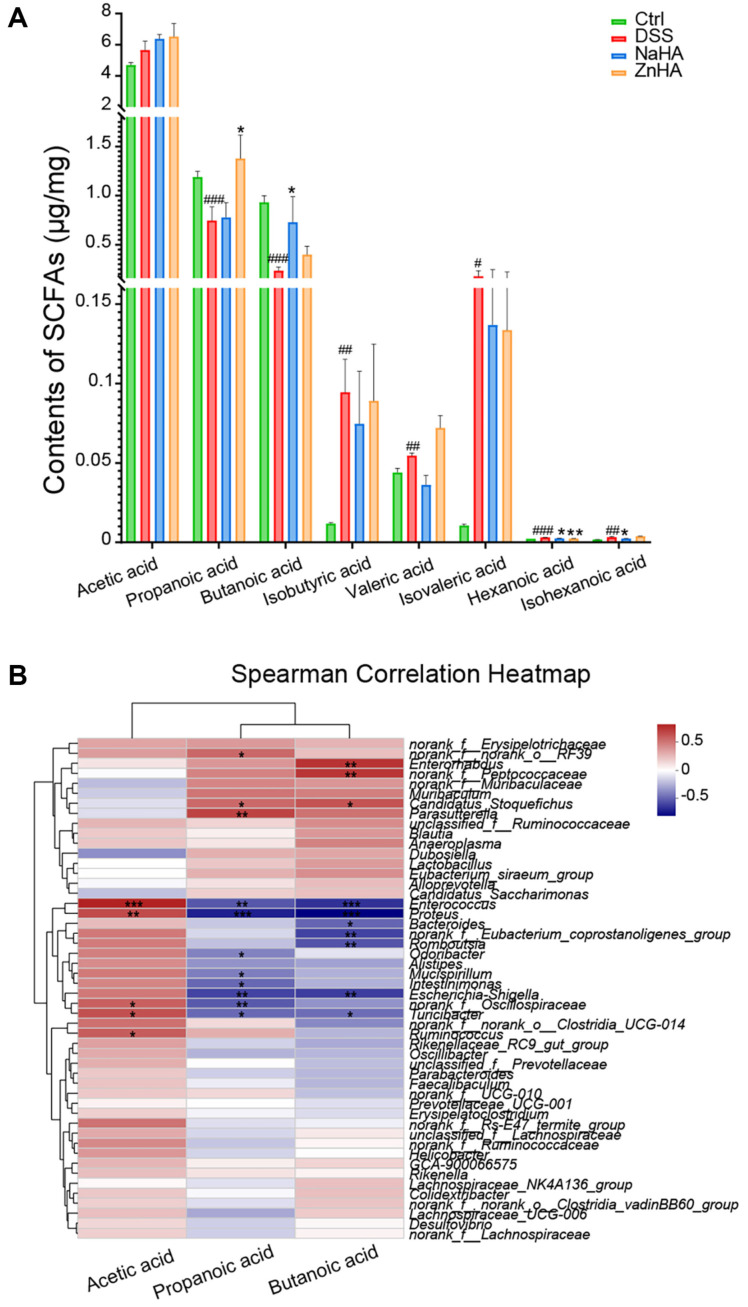
The effects of ZnHA on SCFAs. The contents of SCFAs in mice (**A**) (*n* = 6). Spearman correlation heatmap between SCFAs and intestinal flora (**B**). Data are presented as mean ± SD. ^###^*p* < 0.001, ^##^*p* < 0.01, ^#^*p* < 0.05 versus Ctrl group. ***p < 0.001, **p < 0.01, *p < 0.05 versus DSS group. DSS, dextran sulfate sodium; Ctrl, control; NaHA, sodium hyaluronate; ZnHA, zinc hyaluronate; SCFAs, short-chain fatty acids; SD, standard deviation.

Next, we measured the SCFAs in the colon of each mouse. SCFAs are microbial metabolites produced by the bacterial fermentation of dietary fiber in the intestine [43-45]. The main products include acetic, propionic, and butyric acids. GC-MS was conducted to detect the concentration of several SCFAs, and the data are displayed in Fig. 6A. Following DSS modeling, significant alterations were observed in the SCFAs, including propionic acid, butyric acid, isobutyric acid, and valeric acid, which aligned with findings of previous literature [46]. Administration of ZnHA or NaHA resulted in partial restoration of SCFA levels. Specifically, ZnHA significantly restored propionic acid levels, even slightly surpassing those in the Ctrl group. In contrast, NaHA notably returned butyric acid to levels comparable to those in the Ctrl group. Additionally, ZnHA exhibited a modest capacity to increase butyric acid content. Intriguingly, no discernible differences were observed in acetic acid levels across all groups.

Further correlation analysis (Fig. 6B) showed that propionic acid was positively correlated with *Parasutterella*, *norank_f__norank_o__RF39* and *Candidatus_Stoquefichus* and negatively correlated with *Enterococcus*, *Proteus*, *Escherichia-Shigella*, *Odoribacter*, *Mucispirillum*, *Intestinimonas*, *norank_f__Oscillospiraceae*, and *Turicibacterc*. Butyric acid was positively correlated with *Enterorhabdus*, *norank_f__Peptococcaceae*, and *Candidatus_Stoquefichus* and was negatively correlated with *Enterococcus*, *Proteus*, *Bacteroides*, *norank_f__Eubacterium_coprostanoligenes_group*, *Romboutsia*, *Turicibacter*, and *Escherichia-Shigella*.

## Discussion

As an inflammatory disease, UC often causes intestinal barrier damage, intestinal microbial imbalance, decreased SCFA levels, and immune disorders. For example, berberine can maintain the intestinal mucosal barrier by regulating the Wnt/β-catenin pathway and restore intestinal mucosal immune homeostasis, thereby repairing colitis [47]. After treatment of UC with *Crataegus pinnatifida* polysaccharides, *Alistipes* and *Odoribacter* were found to be significantly enriched in the intestinal tract. *Alistipes* and *Odoribacte* promote the production of propionic acid and acetic acid, exert anti-inflammatory effects, and effectively alleviate UC [48]. Mulberry anthocyanins alleviated colonic tissue damage and the inflammatory reaction in DSS-induced colitis mice and maintained the intestinal barrier by restoring the levels of intestinal tight junction (TJ) proteins (claudin-3, occludin, and ZO-1). In addition, mulberry anthocyanins inhibit DSS-induced intestinal dysbacteriosis [49].

HA is the key constituent of the extracellular matrix in most tissues and can promote the proliferation of probiotics and affect the immune defense of the host. Therefore, we wanted to further explore the specific mechanism by which ZnHA exerts an anti-UC effect to provide a good basis for the subsequent use of HA in UC.

In this study, an acute UC mouse model was constructed by adding DSS to drinking water. Mice with UC show symptoms such as weight loss, fecal thinning, and fecal bleeding, and the DAI score increases significantly, which is consistent with results of a previous report [50]. At the same time, the colon length of mice with UC was significantly shortened, the colon tissue was seriously damaged, and the colon mucosa was significantly thinner in the present study [51]. After the administration of ZnHA, the above adverse symptoms were significantly alleviated, and the symptoms of the mice resolved. At the same time, ZnHA also effectively alleviated the changes in inflammatory factors TNF-α, IL-6, IL-10 and MPO, and played an anti-inflammatory role.

The intestinal epithelium has an efficient barrier function that is mainly mediated by TJ proteins. Currently, TJ proteins mainly belong to the ZO-1, occludin, claudin families [52]. These proteins play a vital role in sustaining the normal barrier by controlling the passive transport of ions and small molecules in epithelial and endothelial cells and forming a barrier through tight junctions [53]. TJ proteins are essential for signal transduction and the regulation of gene expression [54]. As barrier-forming proteins, disorders of claudins may lead to increased intestinal permeability and susceptibility to gut infection in patients with IBD [55]. In addition, ZO-1 plays an important role in Wnt signaling and mitotic spindle orientation [55]. The expression of ZO-1 can promote the repair of the intestinal epithelial mucosa in mice, and the lack of ZO-1 in mice seriously impairs mucosal repair [56]. Simultaneously, abnormalities related to epithelial and endothelial barrier dysfunction occur in occludin-knockout mice, confirming that occludin can effectively regulate intestinal barrier function [57]. Here, we found that the expression of ZO-1 and occludin in the colon of mice was significantly decreased after DSS-induced UC, whereas the concentration of ZO-1 and occludin was significantly increased after the administration of ZnHA. This indicates that ZnHA functioned to repair the intestinal barrier. Through comparison, we have found that ZnHA outperforms NaHA in terms of its anti-inflammatory effect and maintenance of the intestinal barrier, which may be attributed to the unique zinc ion in ZnHA.

Studies have shown that the intestinal microbiota of IBD patients is significantly different from that of healthy people [58]. There are many ways to treat UC by regulating microbes, including probiotics, prebiotics, antibiotics, and FMT [59-61]. In this study, we observed significant changes in the gut microbiota of mice after DSS administration. Furthermore, following treatment with ZnHA and NaHA, there were notable differences in β-diversity compared with the DSS group. This suggests that ZnHA and NaHA may alter the expression of certain microorganisms in the guts of DSS-treated mice. For example, the relative abundance of beneficial microorganisms, such as *Akkermansia*, *Helicobacter*, *Enterorhabdus*, and *Anaerotruncus*, was the highest in the intestinal contents of mice in the ZnHA group.

SCFAs are organic fatty acids with a carbon atom number less than 6, which are produced by microbial fermentation in the intestine and participate in various metabolic processes in the human body [62]. Acetate is the most abundant SCFA in the gut produced from acetyl-CoA derived from glycolysis [63]. Yet, this study found that there was no significant difference in the acetic acid content among the various mice groups. This suggests that it was primarily other SCFAs that played the major regulatory role here. After administration of ZnHA, the propionic acid content was significantly restored. Propionic acid, an abundant SCFA in the intestine, is mainly produced by members of *Bacteroides* [64]. The dominant bacterial genera *Bacteroides* and *Ruminococcus* in the ZnHA group participate in the succinate pathway to produce succinate, which is further converted into propionate by bacteria such as those in the Negativicutes class, thereby promoting propionate synthesis [65-67]. Propionate alleviates inflammatory bowel disease by inhibiting M1 polarization of macrophages [68]. Additionally, the probiotic *Ruminococcus* stabilizes the intestinal barrier [69]. According to literature reports, propionate concentration positively correlates with the abundance of *Parasutterella* [70, 71], which aligns with our correlation analysis results (Fig. 6). In addition, *Akkermansia* is a key mucin-degrading organism that produces propionate and is closely related to propionate metabolism [72, 73]. We speculate that ZnHA promotes the production of propionic acid and restores the intestinal barrier by altering the intestinal flora.

Simultaneously, the administration of NaHA significantly elevated butyric acid levels in the intestines of mice with UC. As one of the most crucial metabolites produced by intestinal microbial fermentation, butyrate possesses various physiological functions, including enhancing intestinal barrier and mucosal function [74-76]. Notably, *Lachnopiraceae* bacteria, representing the dominant flora in the NaHA group, are recognized for their ability to produce butyrate [72]. Furthermore, bacteria such as *Butyricicoccus*, *Prevotellaceae_UCG-001*, and *Lachnospiraceae_ NK4A136_group* exhibited the highest relative abundance in the NaHA group, facilitating butyrate production [77] and aiding in intestinal injury repair. Herein, we speculate that NaHA uses the potent repair properties of butyrate to restore the intestinal barrier, thereby exerting a therapeutic effect on UC.

In summary, ZnHA inhibits inflammation in mice with DSS-induced UC and promotes the formation of SCFAs by modulating intestinal microbiota, which in turn enhances the expression of TJ proteins, maintaining intestinal barrier integrity and aiding in the treatment of UC. However, this study did not further elucidate the pivotal roles of intestinal bacteria such as *Bacteroides* and *Lachnopiraceae* in UC treatment. Additionally, the role of SCFAs in maintaining the intestinal barrier was not determined. Future work will verify SCFA receptors, including GPR41, GPR43, and GPR109A, and examine their interactions with SCFAs. This study provides a solid foundation for the use of ZnHA in UC treatment and facilitates its further development and application.

## Supplemental Materials

Supplementary data for this paper are available on-line only at http://jmb.or.kr.



## Figures and Tables

**Table 1:** Disease activity index (DAI).

Body weight loss (a)	Stool trait (b)	Hematochezia (c)	Score
No	Normal	Normal	0
1–5%	Soft but still formed	Slight bleeding	1
5–10%	Soft	Minor bleeding	2
10–20%	Very soft; wet	Moderate bleeding	3
>20%	Watery diarrhea DAI = (a + b + c) / 3	Bloody stools	4
