# Supplementary material for: Treatment of Acute Ulcerative Colitis with Zinc Hyaluronate in Mice
Source: J Microbiol Biotechnol. 2025 Feb 14;35:e2408050. doi: 10.4014/jmb.2408.08050 (PMC11876020; doi:10.4014/jmb.2408.08050)
Supplement: Supplementary file 1 [file jmb-35-e2408050-supple.pdf]

J. Microbiol. Biotechnol. 2025. 35: e2408050

https://doi.org/10.4014/jmb.2408.08050

Supplementary Figure

1 Methods

All mice were acclimatised for 10 days prior to the initiation of the experiment. Before the formal experiment, a preliminary study was performed to compare the effects of distinct doses of ZnHA (25 and 50 mg/kg) and NaHA (25 and 50 mg/kg) on UC. The optimal doses of ZnHA and NaHA for the treatment of UC were determined using preliminary pharmacodynamic experiments.

All mice were randomly divided into Ctrl, DSS, 5-ASA, NaHA (25, 50 mg/kg), and ZnHA (25, 50 mg/kg) groups. Starting from the first day of the experiment, except for the control (Ctrl) group, other mice drank 2.0% DSS solution freely for 7 days for acute UC. Intragastric administrations were conducted daily, lasting for 8 days from the onset of modelling. The Ctrl and DSS groups were administered normal saline by gavage at a dose of 10 mL/kg. The 5-ASA groups were administered 50 mg/kg by gavage. The NaHA (25, 50 mg/kg), and ZnHA (25, 50 mg/kg) groups were administered 25 or 50 mg/kg by gavage. During the experiment, the diet, activity, and hair status of the mice were observed daily. Body weight, stool traits, and haematochezia were recorded daily. At the same time, the disease activity index (DAI) of mice was calculated. On the 9th day, all mice were euthanised.

After measuring the colon length, the samples were stored at -80°C for subsequent analysis of other indicators. Additionally, the collected spleens were weighed, and the

spleen index was calculated.

Some colon proteins were extracted by homogenising with phosphate buffer saline, and cytokines (TNF-α, IL-6) in the colon were determined using the ELISA reagents and common kit. All experimental procedures were performed in accordance with the manufacturer's guidelines.

2 Results

After establishing the UC model, the effects of different doses of ZnHA and NaHA (25 and 50 mg/kg) were compared (**Supplementary Fig. 1**). As shown in **Supplementary Fig. 1**A–E, we found that 50 mg/kg ZnHA and 50 mg/kg NaHA exhibited better therapeutic effects in UC. These doses significantly alleviated the DAI scores and reduced the expression of inflammatory factors.

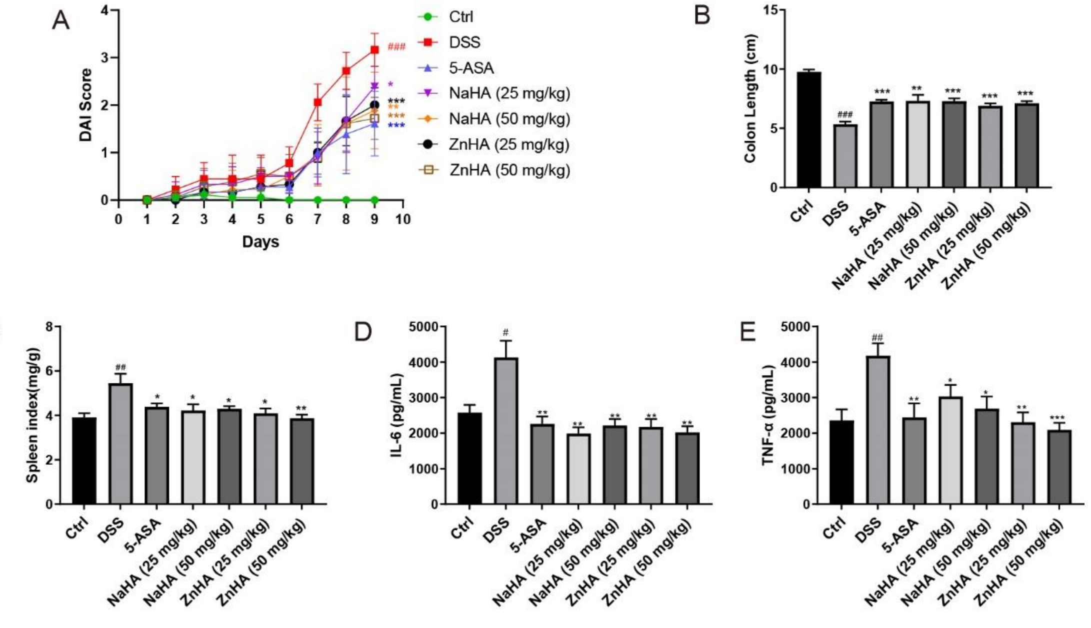

Supplementary Fig. 1. The effect of different dosages of ZnHA and NaHA on DSS-induced acute UC in mice. The effects of different doses of ZnHA and NaHA on UC mice: DAI score (A); colon length (B); spleen index (C); the contents of IL-6 (D) and TNF-α (E) in the colon.
